# Adjustable Thermo-Responsive, Cell-Adhesive Tissue Engineering Scaffolds for Cell Stimulation through Periodic Changes in Culture Temperature

**DOI:** 10.3390/ijms24010572

**Published:** 2022-12-29

**Authors:** Ketpat Vejjasilpa, Iram Maqsood, Michaela Schulz-Siegmund, Michael C. Hacker

**Affiliations:** 1 Pharmaceutical Technology, Institute of Pharmacy, Medical Faculty, Leipzig University, Eilenburger Str. 15A, 04317 Leipzig, Germany; 2 Riphah Institute of Pharmaceutical Sciences (RIPS), Riphah International University (RIU), Lahore 54000, Pakistan; 3 Department of Pharmaceutical Sciences, University of Maryland School of Pharmacy, 20 N Pine Street, Baltimore, MD 21201, USA; 4 Institute of Pharmaceutics and Biopharmaceutics, Heinrich-Heine University, Universitaetsstrasse 1, 40225 Duesseldorf, Germany

**Keywords:** thermally responsive biomaterial, stimulus-responsive biomaterial, mechanical stimulation, smart hydrogel, dynamic light processing, photo-polymerization

## Abstract

A three-dimensional (3D) scaffold ideally provides hierarchical complexity and imitates the chemistry and mechanical properties of the natural cell environment. Here, we report on a stimuli-responsive photo-cross-linkable resin formulation for the fabrication of scaffolds by continuous digital light processing (cDLP), which allows for the mechano-stimulation of adherent cells. The resin comprises a network-forming trifunctional acrylate ester monomer (trimethylolpropane triacrylate, or TMPTA), *N*-isopropyl acrylamide (NiPAAm), cationic dimethylaminoethyl acrylate (DMAEA) for enhanced cell interaction, and 4-acryloyl morpholine (AMO) to adjust the phase transition temperature (T_trans_) of the equilibrium swollen cross-polymerized scaffold. With glycofurol as a biocompatible solvent, controlled three-dimensional structures were fabricated and the transition temperatures were adjusted by resin composition. The effects of the thermally induced mechano-stimulation were investigated with mouse fibroblasts (L929) and myoblasts (C2C12) on printed constructs. Periodic changes in the culture temperature stimulated the myoblast proliferation.

## 1. Introduction

Mechanical stimulation during the cultivation of tissue-engineered constructs can promote tissue development and quality [1]. Different techniques have been applied to exert external stimulation, such as compressive loading systems [2], longitudinal stretch systems that provide uniaxial tension, [3] systems utilizing substrate bending, [4,5] bi-axial traction systems, [6,7] and shear stress input systems [8,9]. However, those systems require complex bioreactor setups to create a suitable microenvironment. [10] We propose an alternative strategy for exerting mechanical stimulation on adherent cells via a stimulus-responsive scaffold. Such a strategy requires a smart biomaterial that changes its swelling degree upon an environmental stimulus that is compatible with the cell culture and that maintains interaction with adherent cells in both the swollen and the de-swollen state. Different stimuli to initiate such a phase transition have been investigated, and the temperature remains the most thoroughly investigated trigger that has been used for the design of various multifunctional materials [11,12,13,14,15,16,17,18]. Based on previous expertise, an *N*-isopropylacrylamide (NiPAAm)-based thermo-responsive material is envisioned here [19,20]. As it has been reported that mammalian cells can be cultured between 30 °C and a physiological temperature without impeding development [21,22,23], a target range for the transition temperature of 30–36 °C was set. The lower critical solution temperature (LCST) of NiPAAm-based materials can be adjusted through copolymerization, whereby hydrophobic comonomers decrease the transition temperature and hydrophilic comonomers have the opposite effect [24,25,26]. PolyNiPAAm has been used in various applications, such as smart surfaces, from which a cultivated tissue layer can be harvested without trypsinization [27]. When the temperature is decreased below a lower critical phase transition temperature (LCST), the pNiPAAm surface layers swell and the cultured cell layer, together with the deposited extracellular matrix, detaches. In order to utilize a thermo-responsive material for cell stimulation, it is necessary that the cells remain adhered to the material body both above and below the transition temperature. We hypothesize that cell adhesion can be sufficiently mediated by introducing positive charges to the material network [28,29]. Hence, we investigate whether this strategy can also be utilized for cell mechanical stimulation through the increased/decreased swelling of the network upon cyclic temperature changes around the LCST with a copolymerized thermo-responsive network comprising a cationic comonomer.

To this end, a resin formulation for the three-dimensional printing of the tissue engineering scaffolds that exhibits thermo-responsive behavior in the targeted temperature range and maintains interaction with adhered cells in the swollen and de-swollen state is warranted. Trimethylolpropane triacrylate (TMPTA) is known as a biocompatible trifunctional macromer and is used as a cross-linker for the formation of an insoluble network of copolymerized NiPAAm [30]. The cationic comonomer dimethylaminoethyl acrylate (DMAEA) is introduced as the functional component to mediate cell adhesion independent of the swelling degree of the material. 4-acryloylmorpholine (AMO) is selected to adjust the transition temperature (T_trans_) of the copolymer network [14].

This work focused on UV light-induced resin copolymerization, first in bulk and thereafter by cDLP printing, and the characterization of the resulting networks with regard to transition temperature and material swelling. Continuous DLP is one of many three-dimensional printing techniques that have been used for medical scaffold fabrication with high resolution [31,32]; it uses a photo-curable resin that is polymerized in layers by projected light. However, not many biocompatible resins for photo-polymerization are available [33,34,35]. In this work, we additionally introduce tetrahydrofurfuryl alcohol polyethylene glycol ether (glycofurol, GF) as an alternative solvent for resin formulation. GF has already been utilized as a biocompatible solvent or diluent for drug delivery to both human and animal tissue. Cytocompatibility of the resin-derived networks is evaluated with L929 and C2C12 cells. Promising formulations are cultivated and the response of the adherent cells to the periodic changes in cultivation temperature are compared.

## 2. Results and Discussion

**Figure 1:**
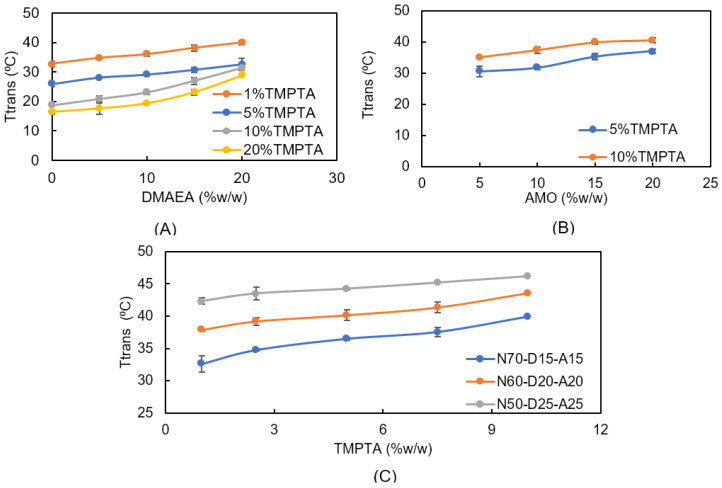
Transition temperatures of photo-polymerized discs as obtained by DSC. (**A**) Resins with 1–20% *w*/*w* TMPTA, 0–20% *w*/*w* DMAEA, and 0% *w*/*w* AMO. (**B**) Effect of AMO on transition temperature (5–10% *w*/*w* TMPTA, 15% *w*/*w* DMAEA). (**C**) Effect of TMPTA (1–10% *w*/*w*) on transition temperature in different resin formulations (N70-D15-A15, N60-D20-A20, and N50-D25-A25).

Discs and a three-dimensional scaffold from thermo-responsive networks composed of NiPAAm, DMAEA, TMPTA, and AMO were successfully polymerized by photo-induced bulk polymerization and cDLP, respectively. In order to identify a suitable resin formulation, different series of monomer mixtures were cross-polymerized into nonporous discs by photo-induced bulk polymerization. The transition temperatures of the water-swollen discs were quantified by DSC, as presented in Figure 1. All the DSC analyses were performed in aqueous solutions. Although the transition temperature is generally affected by osmolarity, pH value, electrolyte type, and the concentration of the solution [36], we did not intend to simulate the exact cell culture conditions with the medium serum and additives to obtain a clearer signal only affected by the polymer network composition. It was found that the cationic monomer DMAEA and the network former TMPTA significantly influenced the transition temperature, and the NiPAAm-based formulations with transition temperatures ranging from 16.4 °C ± 0.9 °C (0% *w*/*w* DMAEA, 1% *w*/*w* TMPTA) to 40.0 °C ± 0.7 °C (20% *w*/*w* DMAEA, 20% *w*/*w* TMPTA) were obtained (Figure 1A). When the samples with the same TMPTA content were compared, the DMAEA acted as hydrophilic comonomer and an increasing content resulted in an increasing T_trans_. For example, within the 5% *w*/*w* TMPTA discs the transitional temperature increased from 25.9 °C ± 0.8 °C (0% *w*/*w* DMAEA) to 32.5 °C ± 2.0 °C (20% *w*/*w* DMAEA). Looking at the TMPTA effect in the absence of DMAEA, the transition temperature considerably decreased from 32.7 °C ± 0.9 °C (0% *w*/*w* DMAEA, 1% *w*/*w* TMPTA) to 16.4 °C ± 0.8 °C (0% *w*/*w* DMAEA, 20% *w*/*w* TMPTA). An increase in the feed ratio of TMPTA created denser networks, thus decreasing the distance between the polymer segment chains due to the trivalent chemistry of the monomer which directly affected hydration capacity, subsequently decreasing the material’s transition temperature [37].

Both comonomers, DMAEA and TMPTA, affect the transition temperature of the NiPAAm-based networks, but both monomers also change other key characteristics of the material, i.e., the charge and cross-linking density. With the implementation of a hydrophilic but functionally inert monomer, 4-acryloylmorpholine (AMO), we intended to further adjust the transition temperature of the cross-polymerized networks to the desired range of 33 °C to 36 °C without changing the charge and cross-linking. The molecular character of the morpholine residue attracts water molecules into the polymeric network and promotes an increase in material swelling [14,38,39]. In addition, AMO was also expected to promote the solubility of the resin in less hydrophobic and more biocompatible solvents. As illustrated in Figure 1B, the presence of AMO in the TMPTA-cross-linked copolymer increased the transitional temperature. For example, the discs copolymerized from 5% *w*/*w* TMPTA, 15% *w*/*w* DMAEA, and 5% *w*/*w* AMO had a T_trans_ of 30.7 °C ± 1.7 °C. The transition temperatures gradually increased to 37.1 °C ± 0.6 °C (5% *w*/*w* TMPTA, 15% *w*/*w* DMAEA, 20% *w*/*w* AMO) with the increasing AMO content. The resin composition was optimized at a DMAEA content of 15% (*w*/*w*), which was the best compromise of a high amine content and cell survival in the initial compatibility tests. With regard to the desired phase transition temperature, we identified promising formulations from the data presented in Figure 1 that consist of 1–5% *w*/*w* TMPTA, 10–15% *w*/*w* DMAEA, and 1–15% *w*/*w* AMO. These formulations were investigated in more detail.

### 2.1. Swelling Characteristics of Photo-Polymerized Discs

**Figure 2:**
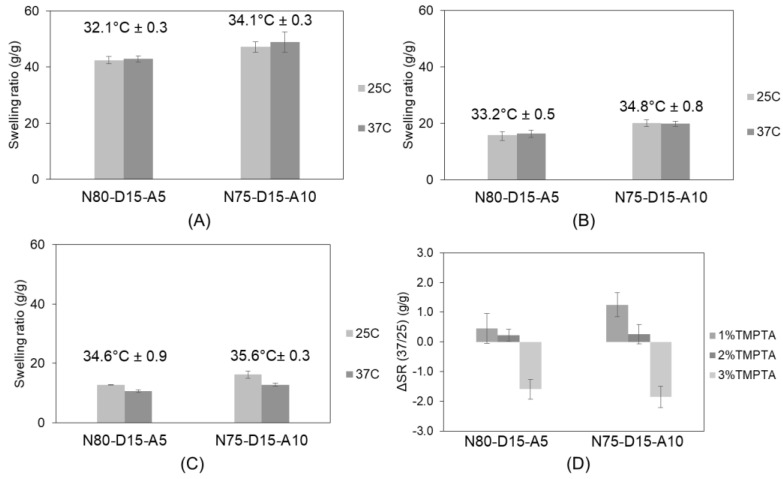
Swelling ratio at different temperatures and T_trans_ of 1% TMPTA (**A**), 2% TMPTA (**B**), 3% TMPTA cross-linker (**C**), and ΔSR (37/25) of 1%, 2%, and 3% TMPTA cross-linker (**D**). Negative value indicates shrinkage of sample discs.

We postulate that mechanical stress will be exerted by the dimensional changes in the materials as a result of different material water contents in response to the changes in the environmental temperature. Consequently, the swelling ratios of the different resin compositions were evaluated at 25 °C and 37 °C (Figure 2). A more specific focus was given to the transition temperatures of the formulations with 5% and 10% *w*/*w* AMO and low contents of the network-forming comonomer TMPTA (1%, 2%, and 3% *w*/*w*). The discs with 10% *w*/*w* AMO and 15% *w*/*w* DMAEA (N75-D15-A10) had significantly higher transitional temperatures than the formulations with 5% *w*/*w* AMO (N80-D15-A5) for all the TMPTA additions. The discs with 1% *w*/*w* TMPTA (15% *w*/*w* DMAEA and 5% *w*/*w* AMO, Figure 2A) had the lowest T_trans_ at 32.1 °C ± 0.3 °C, which was increased to 34.6 °C ± 0.9 °C by increasing the AMO content to 10% *w*/*w*.

We also observed a clear change in the swelling ratios at the investigated temperatures of 37 °C and 25 °C when different TMPTA concentrations were compared. In order to show if water is expelled from the formulation by thermally induced gelation, we defined the parameter ΔSR (37/25) (as defined in the methods section), which becomes negative if the phase transition occurs between the two temperatures and the cross-linked networks expel water, while positive values indicate an increase in swelling at 37 °C. The discs with 3% TMPTA and 10% *w*/*w* (N75-D15-A10) or 5% *w*/*w* (N80-D15-A5) AMO successfully shrank by −1.2 ± 0.4 g/g and −1.0 ± 0.1 g/g. In contrast, the lower TMPTA contents resulted in positive values for ΔSR (37/25) as a result of a reduced cross-linking density. We also observed the expected effect of AMO on network hydrophilicity [40].

### 2.2. Three-Dimensional Scaffold Fabrication

From the described resin formulations that were processed into disc-shaped samples by the bulk photo-polymerization, selected formulations (3% *w*/*w* TMPTA) were transferred to processing by a three-dimensional printer using the concept of dynamic light processing (DLP). We investigated formulation parameters that are specific to the applied three-dimensional printing technique, namely exposure time per layer, the photo-initiator content (PI), and the solvent, as these parameters have been reported before to influence printing quality and the scaffold’s mechanical properties [41,42]. The selected monomer formulations (3% *w*/*w* TMPTA and 15% *w*/*w* DMAEA) with an optimized T_trans_ (0% AMO: 34.6 °C ± 0.9 °C and 5% AMO: 35.6 °C ± 0.3 °C) determined via disc polymerization were prepared and diluted with ethanol to yield a concentration of 10% (*w*/*v*). Two different scaffold designs, namely a raft and a three-dimensional lattice, were printed (Figures S1 and S2). The raft-configuration scaffolds were selected to investigate the biocompatibility of the material as the geometry allows the observation of the cells on the cell impermeable scaffold surface. Cell proliferation can be conveniently observed by microscopy. The second design is a macroporous lattice of 7.6 × 7.8 × 2.5 mm (length × width × height), with fiber diameters of 240 µm. A fiber arrangement that used thicker fibers (470 µm) as the scaffold foundation (first layer) was used. In the subsequent nine fiber layers, the fiber orientation was rotated by 90° per layer. The scaffolds (lattice and raft) were composed of 50 layers with a thickness of 50 µm each. The macroporous structure of the lattice was selected to enable cell adhesion and proliferation and allow for efficient nutrient exchange via interconnecting channels [43,44]. The resins were printed with various exposure times per layer (10, 20, and 30 s).

**Figure 3:**
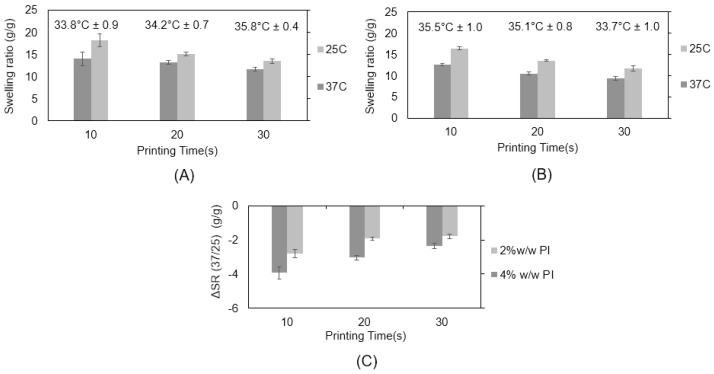
Swelling ratio (g/g) of 3% TMPTA N80-D15-A5 scaffold with (**A**) 2% *w*/*w* and (**B**) 4% *w*/*w* photo-initiator (Irg819). Scaffold T_trans_ values as determined by DSC added to the graphs. (**C**) ΔSR (37/25) values of the presented formulations.

The intended property of the materials presented here is to respond to temperature and to exert a mechanical stimulus on adherent cells by volume change. The type and concentration of PI as well as the exposure time are known to influence the network characteristics [45]. Lower contents of PI and shorter exposure times are expected to yield materials with reduced cross-linking and a smaller response in the swelling change between 25 °C and 37 °C, as expressed by a low value of ΔSR (37/25) (Figure 3). Based on a comparison of the corresponding resin formulations processed into discs (N80-D15-A5), we observed the strongest change in swelling with 4% PI at an exposure time of 10 s (Figure 3C). As for the photo-polymerized discs, ΔSR (37/25) was also compared for different scaffold formulations. We observed no significant change in transition temperature as a result of PI content and exposure time.

### 2.3. Three-Dimensional Scaffold Fabrication with Glycofurol as Solvent

In the experiments using ethanol as a solvent/diluent for cDLP, certain limitations concerning the printability of the resins, such as the premature detachment of the sample and low structural resolution due to low resin viscosity, became apparent. With the objective being to address these issues, glycofurol (GF), which has a higher viscosity than ethanol, was utilized [46,47]. The increased viscosity of GF in comparison to ethanol could potentially improve scaffold resolution during printing due to damping effects [46,48,49]. GF has been introduced as a biocompatible solvent for biomedical applications and as a pharmaceutical excipient. GF in a 50:50-mixture with PBS was administered to rat brain tissue and was well tolerated and provoked only a minor inflammatory response [46]. The possible residual amount of GF that might be released from the washed scaffolds over time is considered to be much less than the concentrations tested in the mentioned study. Therefore, residual GF is not expected to affect the cellular response but a chromatographic evaluation of the amount of residual GF will become necessary in future studies.

**Figure 4:**
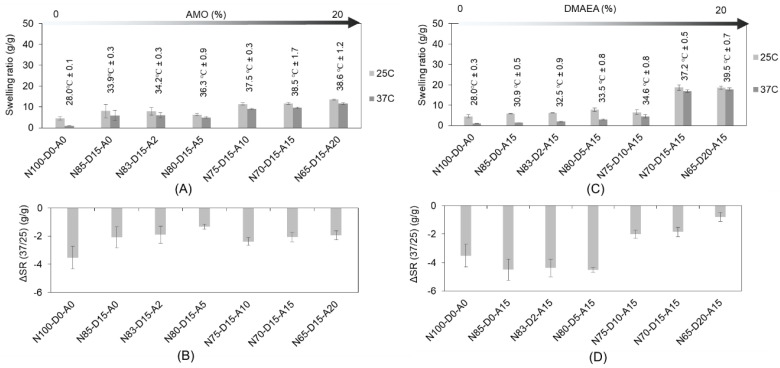
Swelling behavior of three-dimensional scaffold printed from GF-based resins (3% *w*/*w* TMPTA, 4% *w*/*w* PI, printing time 10 s/layer) at different temperatures (25 °C and 37 °C) with the transition temperature with different ratios of (**A**) AMO and (**B**) DMAEA. The corresponding ΔSR (37/25) is shown in charts (**C**,**D**).

It was possible to print GF-diluted resins with preserved thermo-responsive properties and effectively halve the exposure time per printed layer from 20 to 10 s. Furthermore, premature detachment of the construct from the printing head during printing could also be avoided. The swelling ratios of the GF-based scaffold for both geometries (raft and lattice) were determined by varying different DMAEA and AMO ratios at different temperatures (25 °C and 37 °C) (Figure S3). The lattice scaffolds fabricated from resins without DMAEA and AMO (3% TMPTA N100-D0-A0) showed the strongest change in swelling due to a low transition temperature of 28.0 °C ± 0.1 °C, which corresponds to the expectations for a pure cross-linked NiPAAm matrix (Figure 4). As expected, the addition of AMO and DMAEA increased the transition temperature that was established for the ethanol-based resins. The increased AMO content effectively increased the transition temperature of the cross-linked bulks from 33.9 °C ± 0.3 °C (N85-D15-A0) to 38.6 °C ± 1.2 °C (N65-D15-A20). The transition temperature of the scaffolds with different ratios of DMAEA and an AMO content of 15% was elevated from 30.9 °C ± 0.5 °C (N85-D0-A15) to 39.5 °C ± 0.7 °C (N65-D20-A15). The corresponding values of ΔSR (37/25) decreased from (−4.5 ± 0.1) g/g (N85-D0-A15) to (−0.8 ± 0.2) g/g in (N65-D20-A15). The results of ΔSR (37/25) showed the effect of DMAEA, which could increase the transition temperature and stabilize the NiPAAm-based networks from extensive collapse in the investigated temperature range, thus decreasing ΔSR (37/25). From the authors’ perspective, the ΔSR (37/25) values should be measurable and negative but in a moderate range (−3 to −2 g/g) in order to maintain cell adhesion to the biomaterial surface.

### 2.4. Rheological Properties of Scaffolds

Changes in cultivation temperature are expected to switch the scaffold swelling and exert mechanical stimulation, which is an important factor that influences cell fate [50,51]. Rheological characterization of the scaffold moduli by oscillation rheology revealed that the moduli are in the range of the literature data on cellular stiffness (myoblasts: 2 kPa, fibroblasts: 1–10 kPa) [52,53]. As shown in Figure S4, the determined storage moduli of the printed scaffolds increased with the exposure time (S4a) and photo-initiator concentration (S4b). For example, at the transition temperature, the storage moduli of the samples fabricated with 4% PI and 10 s of exposure per layer were 1.2 and 1.5 times higher as compared to the samples with 3% PI and 2% PI, respectively. An increase in exposure time per layer from 10 s/layer to 20 and 30 s/layer doubled the moduli. The transition temperature remained almost unchanged as the altered parameters (concentration of PI and exposure time per layer) do not affect the chemical composition of the network. Network density, however, is affected by these parameters, as observed by the changes in the network swelling. The incorporation of DMAEA strongly decreases the scaffold moduli (Figure S5). At 20% *w*/*w* DMAEA, the hydrophilic contribution of the cationic monomer was strong enough to fully suppress the coil-globule transition of the copolymeric network and no change in storage modulus could be observed any more in the investigated range, which correlates with the observed increase in transition temperature. Apart from N93-D2-A5, the investigated samples (Figure S5) showed very low temperature-induced changes in the bulk storage moduli. In this context, it should be considered that the construct volume change and the corresponding displacement of the focal adhesion points with the cells are the primary causes of the mechanical stress on the adhered cells. Therefore, the correlation between the volume changes that are compatible with the maintenance of cell adherence and cell survival and the storage modulus change in the bulk hydrogel is not obvious and needs further experimental evaluation.

### 2.5. Biocompatibility Evaluation

**Figure 5:**
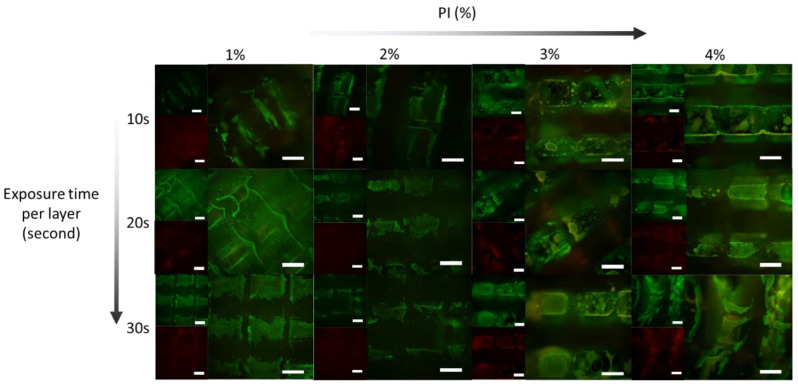
Fluorescence images of L929 fibroblasts on 3% TMPTA, N80-D15-A5 scaffolds (37 °C) at different photo-initiator ratios (horizontal) and exposure times (vertical) after live/dead staining at day 7. In each group, one small picture shows the fluorescence channel for calcein (green) and another the fluorescence wavelength for ethidium homodimer (red). The large picture in each group shows an overlay of both channels. Scale bars represent 500 µm.

We chose a promising, photo-polymerizable thermo-responsive formulation (3% *w*/*w* TMPTA N80-D15-A5, 4% *w*/*w* PI, exposure time 10 s per layer), which has a transition temperature of 36.3 °C ± 0.9 °C and the ΔSR (37/25) of (−1.3 ± 0.2) g/g and shows a change in material modulus from 2.8 kPa (30 °C) to 7.1 kPa (37 °C). Both moduli are within the physiological range of 1–10 kPa and are reported as the best suited for fibroblasts [54]. Therefore, the material is expected to support cell viability at both temperatures, and the periodic change in modulus is expected to stimulate cell proliferation and differentiation. First, we tested the effect of exposure time and PI concentration on the biocompatibility of the resin formulation with murine fibroblasts (L929) at the static cultivation temperature of 37 °C (Figure 5). The cells were stained with calcein-AM and ethidium homodimer on printed scaffolds. All of the formulations showed coverage with live cells at day seven, which, in comparison to the cell density after seeding, indicates a considerable proliferation. There was no discernible difference between the different tested formulations. On a cellular level, the fibroblasts maintained a well-spread morphology. A low number of cells were found to be dead, as indicated by the fluorescence signal in the red channel due to the DNA-bound ethidium homodimer (Figure 5). The diffuse character of the fluorescence signal also indicates some degree of autofluorescence of the materials. The low number of distinctly stained dead cells suggests no explicit material toxicity, but rather an expected turnover of the cells.

**Figure 6:**
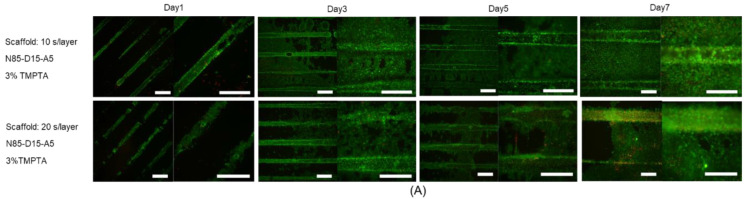

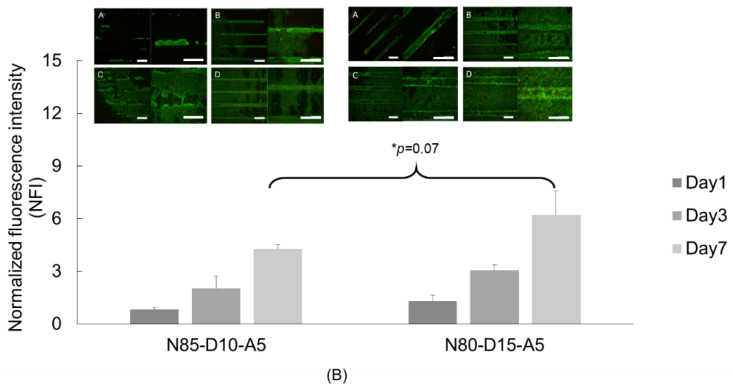
(**A**) Cellularity (L9292 fibroblasts) on GF-based scaffolds with raft architecture (N80-D15-A5, 4% PI, 3% TMPTA) printed with different exposure times per layer (10 and 20 s/layer) at different time points (1, 3, 5, and 7 days). (**B**) Normalized intensities of green fluorescence emitted from live cells on two formulations, N85-D10-A5 and N80-D15-A5 (3% TMPTA, 10 s/layer), at day 1, 3, and 7. Scale bars equal 500 µm (low magnification (4×), left images) or 200 µm (higher magnification (10×), right images). *p*-value is denoted as * *p*.

The scaffolds of the raft architecture, as illustrated in supplementary Figure S1, provide cell adherence in one plane for better microscopic evaluation. The L929 fibroblast viability was microscopically evaluated on scaffolds fabricated from formulation N80-D15-A5 with 3% *w*/*w* TMPTA and 4% *w*/*w* PI, with 10 or 20 s of exposure time per layer. The fluorescence microscopic images of the cells on the scaffolds after cultivation for 1, 3, 5, and 7 days at a constant temperature (37 °C) are summarized in Figure 6. On all the samples, cell proliferation was visible to a comparable degree. No significant difference in compatibility on the scaffolds fabricated with exposure times of 10 and 20 s per layer was visible (Figure 6A). Based on these positive results, the scaffolds of the raft architecture with a DMAEA content of 15% and 10% *w*/*w* were printed with an exposure time of 10 s/layer and evaluated. The normalized intensities of the calcein fluorescence signals (NFI) were plotted and a 1.5-fold (*p* = 0.07) higher NFI was observed for N80-D15-A5 as compared to N85-D10-A5 at day 7 (Figure 6B). We consider this as an indication of the benefit of a content of 15% DMAEA to optimize cell proliferation and survival.

### 2.6. Cellular Response to Scaffolds under Periodic Changes in Cultivation Temperature

**Figure 7:**
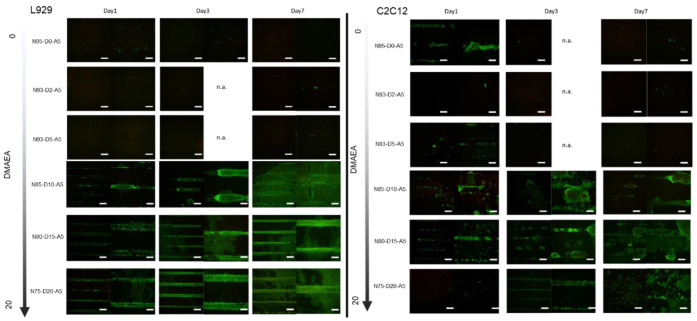
Cellularity of L929 and C2C12 cells on printed scaffolds (3% *w*/*w* TMPTA, 5% *w*/*w* AMO, 4% *w*/*w* PI, exposure time 10 s/layer) in raft architecture cultivated under periodically changed temperature (cycle: 23 h at 37 °C and 1 h at 30 °C). The fluorescent micrographs showed overlaid live/dead staining and were recorded at day 1, 3, and 7. Scale bars represent 500 µm (low magnification (4×), left images) or 200 µm (higher magnification (10×), right images).

The cells were seeded on scaffolds of the raft architecture fabricated from resins with different ratios of DMAEA and were subjected to periodic changes in cultivation temperature in order to demonstrate the scaffold’s ability to sustain cell adhesion above and below transition temperature and to exert mechanical stimulation to the adherent cells. Figure 7 shows cellularity on scaffolds cultivated for 1, 3, and 7 days with a daily reduction in cultivation temperature to 30 °C for one hour. The L929 cells maintained adherence to the scaffolds fabricated from the resins with a DMAEA content of at least 10% *w*/*w*. The scaffolds with less than 10% *w*/*w* DMAEA showed almost no cell attachment. The L929-seeded scaffolds with a higher amount of DMAEA showed a proliferation of adherent cells over the cultivation time of 7 days. Microscopically, the cells tended to initially adhere to the grooves of the scaffold structure, filling the accessible grooves after gravitational seeding. Upon proliferation, the cells grew outward and eventually covered the full surface of the scaffold. Below a threshold value of 10% DMAEA, the cells detached from the scaffolds during the temperature cycles. This confirms the cell adhesive effect of the positively charged moieties in DMAEA. In addition to this chemical effect, more extensive changes in the swelling ratio in the material with a lower transition temperature possibly contributed to the observed cell detachment.

Another cell type that should benefit from mechanical stimulation is the myoblast cell. C2C12 mouse myoblasts have previously been utilized as a model for mechanical stimulation [55,56]. For this reason, we also observed the fate of C2C12 cells on printed formulations. Here, we demonstrated that the C2C12s cultivated on the raft scaffolds at constant temperature (37 °C for up to 7 days) showed almost no cell growth (Figure S6). The scaffolds fabricated from resins N85-D10-A5, N80-D15-A5, and N75-D20-A5, however, showed cell proliferation when cultured under a periodically changed cultivation temperature. Overall, the differences observed between the isothermal and periodically changed cultivation conditions indicate a successful mechanical stimulation of the adherent cells by the material as the mechanical stress from the material swelling is considered to influence and up-regulate molecules such as calmodulin, nNOS, MMP-2, HGF, c-Met, and mitogen-activated protein kinase [51,56].

### 2.7. Effect of DMAEA Content on Cell Proliferation on the Scaffolds

**Figure 8:**
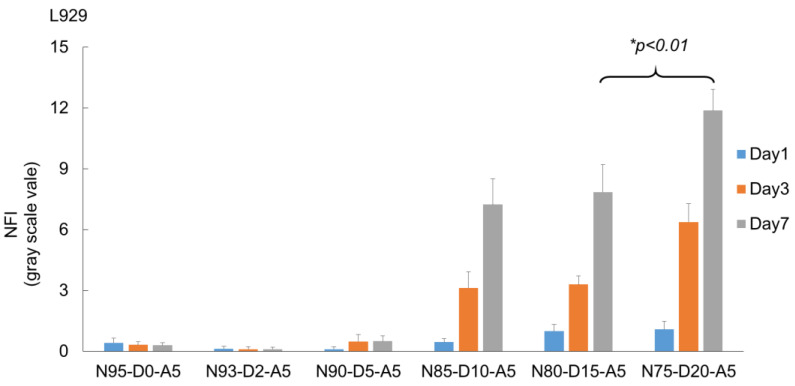

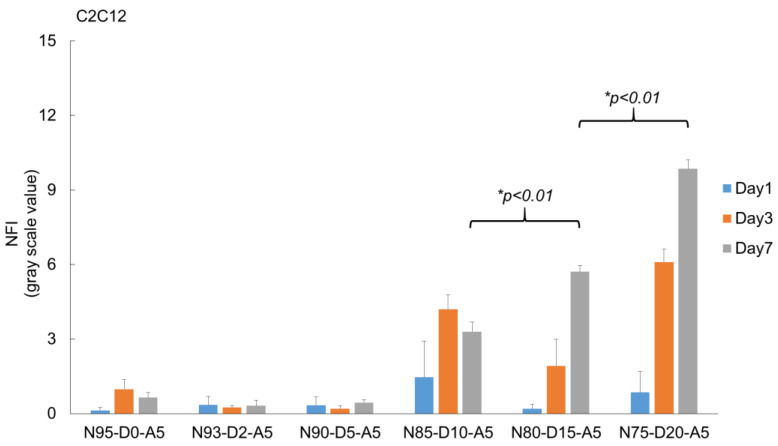
Normalized fluorescence intensity (NFI) at day 1, 3, and 7 of calcein-stained L929 (**top**) and C2C12 cells (**bottom**) on raft scaffolds (3% *w*/*w* TMPTA, 4% *w*/*w* PI, printing time 10 s/layer) of different DMAEA compositions (0–20% *w*/*w*) upon cultivation with periodic changes in cultivation temperature. Statistically significant differences are denoted by *.

Fluorescence intensity was quantified to further characterize the effect of different cationic monomer feeds on the investigated cells (Figure 8). As expected, the materials with a low DMAEA content showed low fluorescence signal intensities. The scaffold with DMAEA feeds of more than 10% in the printing resins yielded promising results. The NFI at day 1 on different formulations showed no statistical difference between the different materials for both cell lines. However, we observed an increase in the average NFI with the increasing DMAEA content. At day 3, the scaffolds with 0–5% DMAEA showed no increase in the intensities, whereas the cells on scaffolds with 10% and more DMAEA showed a significant increase in NFI. The signal intensities of the L929 cells on 10%, 15%, and 20% DMAEA on the raft scaffold increased 9.2-, 10.2-, and 19.8-fold as compared to the cells on scaffolds from resins without DMAEA on the same day. On scaffolds with 15% and 20% DMAEA, the NFI increased steadily over the investigated time points. At day 8, the differences in NFI became more obvious, and the L929 cells on the scaffolds with 20% DMAEA displayed the highest calcein stain (11.9 ± 1.0).

The equivalent experiment with C2C12 myoblasts revealed similar results in response to periodic changes in the cultivation temperature. On scaffolds with less than 5% *w*/*w* DMAEA, cell proliferation was not supported. An interesting behavior of the C2C12 cells was seen on 10% DMAEA scaffolds. The NFI increased from day 1 to 3 but then declined between day 3 and 7. We attribute this to an insufficient number of cationic adhesion moieties to maintain cell adhesion over a larger number of swelling/deswelling cycles. In comparison to the L929 fibroblasts, the C2C12 have been described as more sensitive to material changes [57]. At a constant cultivation temperature, the cellularity of the C2C12 cells on the 15% *w*/*w* DMAEA scaffolds was low and the cells appeared to be predominantly rounded without significant cell–cell contacts (Figure S6). The C2C12 on scaffolds (15% and 20% *w*/*w* DMAEA) cultivated with periodic temperature changes showed proliferation, as indicated by a fold increase in fluorescence intensity of 8.8 (15% DMAEA) and 15 (20% DMAEA) from day 1 to 7.

**Figure 9:**
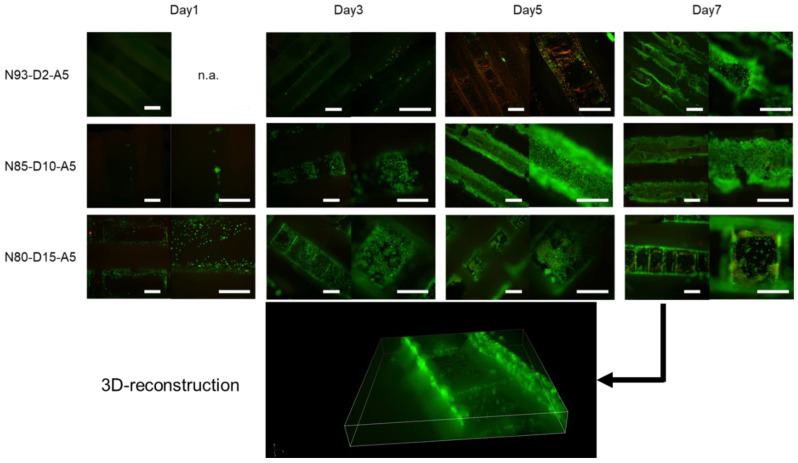
Fluorescence micrographs of three-dimensional lattice-type scaffolds seeded with L929 fibroblasts (2, 10, and 15% *w*/*w* DMAEA, 5% *w*/*w* AMO, 3% TMPTA, 10 s/layer, 4% *w*/*w* PI) and cultivated with periodic temperature changes at different time points (1, 3, 5, and 7 days). Scale bars equal 500 µm (low magnification (4×), left images) or 200 µm (higher magnification (10×), right images). Three-dimensional reconstruction of fluorescent images of N80-D15-A5 at 7 days.

The promising results of the raft-type scaffolds motivated an evaluation of the L929 cells on a lattice-type scaffold that provides a more physiological template for the cell–cell interactions (Figure 9) [58,59]. The resins were processed into lattice-type scaffolds that were designed to allow for effective cell seeding and nutrient distribution through the porous architecture [60,61,62]. At day 1, a low number of cells was observed on the scaffolds with low DMAEA contents in the resins, while the cellularity was increased on the 15% *w*/*w* DMAEA scaffolds, indicating the benefit of cationic moieties for interactions with the polyanionic cell membranes. At day 3, the cells began to spread on the scaffold with at least 10% DMAEA. The NFIs at day 5 and 7 were increased, which suggests cell proliferation within the scaffolds. A reconstruction of the confocal fluorescence microscope slices recorded from the calcein-AM stained L929 cells on a N80-D15-A5 scaffold at day 7 demonstrated high cellularity on the material strands (Figure 9).

Even the scaffolds with low DMAEA (2% *w*/*w*) showed cell proliferation and good cell viability at day 7, but the DMAEA contents of 10% and 15% again proved more effective. A possible explanation for the increased cell survival on the three-dimensional porous scaffold could be the more physiological microenvironment generated during three-dimensional cultivation [58,59,63,64].

In this study, the specific focus was to identify a material that would a show phase change shortly below physiological temperature and exhibit a moderate change in swelling while incorporating functional groups that mediated cell-adhesion above and below the phase transition temperature. This property is not inherent to conventional thermally responsive materials but key to our intended use as tissue engineering scaffold material that could exert a mechanical stimulation on cells cultivated on the material under periodic changes in cultivation temperature encompassing the transition temperature of the material. Material formulations are presented that realize these properties. The effects of the swelling changes on the proliferation and survival of the fibroblasts and myoblasts are shown. With the material development successfully demonstrated in this study, subsequent work will have to elucidate the extent and frequency of the mechanical stimulation and the cellular effects in comprehensive detail.

## 3. Materials and Methods

### 3.1. Materials

*N*-Isopropylacrylamide (NiPAAm) (Tokyo Chemical Industry [TCI], Tokyo, Japan), hexane (Sigma-Aldrich), trimethylolpropane triacrylate (TMPTA) (Sigma-Aldrich), dimethylaminoethyl acrylate (DMAEA) (Sigma-Aldrich, Taufkirchen, Germany), 4-acryloylmorpholine (AMO) (Sigma-Aldrich), phenylbis(2,4,6-trimethylbenzoyl)phosphine oxide (BAPO or Irgacure819) (Sigma-Aldrich), absolute ethanol (EtOH) (Sigma-Aldrich), tetrahydrofurfuryl alcohol polyethylene glycol ether (glycofurol or GF) (Sigma-Aldrich), Dulbecco’s phosphate buffered saline (PBS) (Sigma-Aldrich), calcein-AM (Thermo Fisher Scientific, Waltham, MA, USA), ethidium homodimer (EthD-1) (Thermo Fisher Scientific), low-glucose Dulbecco’s modified Eagle’s medium (DMEM) (Merk), penicillin streptomycin (pen/strep) (Gibco, Thermo Fisher Scientific, Waltham, Massachusetts, USA), and fetal bovine serum (FBS) (Sigma-Aldrich) were purchased and used as received. L929 fibroblasts and C2C12 myoblasts were purchased from CLS Cell Lines Service GmbH (Eppelheim, Germany).

The NiPAAm was purified before use. In brief, the NiPAAm was dissolved in dried warm hexane under constant magnetic stirring. The solution was placed at −20 °C to encourage recrystallization of the NiPAAm. The recrystallized NiPAAm was then separated with a cylindrical funnel and washed again with cold hexane and dried under reduced atmospheric pressure for 72 h.

### 3.2. Methodology

*Nomenclature.* The resin formulations are represented as x% NiPAAm, y% DMAEA, and z% AMO (all *w*/*w*) and labeled by the code Nx-Dy-Az. The intended amount of TMTPA was added to the monomer solution as exemplarily described here: the resin 3% TMPTA 80N-15D-5A was prepared by mixing 80% *w*/*w* NiPAAm, 15% *w*/*w* DMAEA, and 5% *w*/*w* AMO. To this mix, 3% *w*/*w* TMPTA was finally added with respect to the mass of the monomer solution. In a typical batch, such a resin mix would finally be composed of 2.0 g NiPAAm, 0.39 g DMAEA, 0.11 g AMO, and 0.075 g TMPTA. All the formulations are summarized in Tables S1 and S2 in the Supplementary Materials.

**Figure 10:**
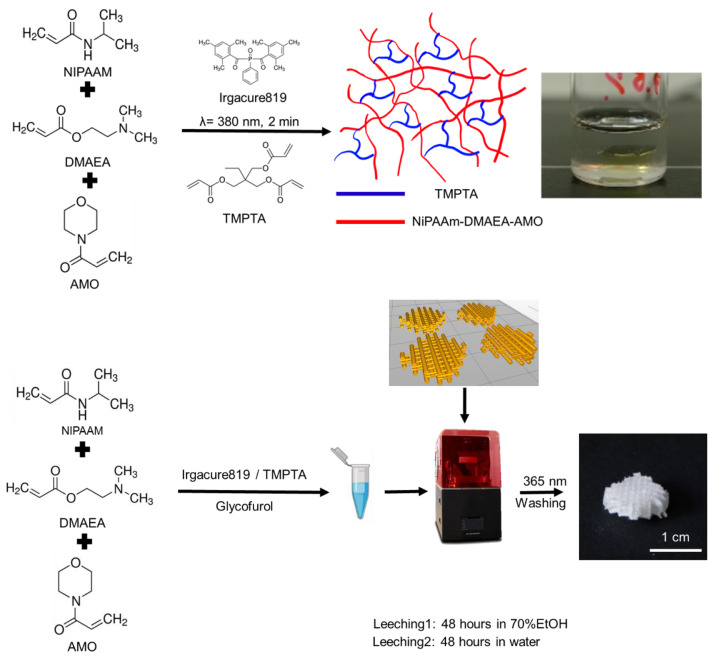
Stimuli-responsive fabrication of temperature-sensitive biomaterials by photo-induced bulk polymerization and cDLP printing. A monomer mixture with PI is irradiated with UV light to cross-link the resin in disc shape or as scaffold produced by cDLP. The cross-linked matrices were washed with aqueous ethanol (70% (V/V)) and water.

*Synthesis of hydrogel discs by photo-induced polymerization.* In order to test the photo-induced copolymerization of the monomer mixtures and investigate the effects of the comonomer ratios (Table S1) on the material properties before three-dimensional fabrication, the resins were prepared by vigorously mixing the corresponding ratios of monomers in ethanol to obtain a concentration of 10% (*w*/*v*) with 4% (*w*/*w*) photo-initiator (PI, Irgacure819) relative to the mass of the monomer mix. A commercial UV chamber (Melody Susie Violet II, Model DR-301C, 36W) was used for photo-cross-linked disc fabrication. Briefly, 200 µL of the monomer-PI-solution was pipetted into caps of polypropylene microcentrifuge tubes (Eppendorf, Germany) and exposed to UV light (380 nm) for two minutes. The cross-linked discs were removed from the caps and washed with diluted ethanol (70%) and distilled water for 24 h each (Figure 10).

*Three-dimensional printing of tissue engineering scaffold*. A commercial cDLP three-dimensional printer (PICO2 HD27 UV, Asiga, Erfurt, Germany) was used for three-dimensional printing. The monomer mix (Table S2) was prepared as described above and subsequently dissolved with either ethanol or glycofurol at a ratio of 2:1 and finally transferred into a building tray, of which the volume was reduced by a customized silicon and Teflon insert. The scaffolds were printed at room temperature with a light intensity of 36.0 mW·cm^−2^ with a layer thickness of 50 µm. The scaffold structures (raft and lattice) (Figure S1) were constructed by the Composer program (Version 1.2.5, Asiga). The exposure time per layer (10, 20, and 30 s per layer) was varied along with the different ratios of photo-initiators. The freshly printed scaffolds were immersed in ethanol (70%) for 48 h to extract solvents and unreacted monomers and subsequently in distilled water or culture medium for further use.

*Swelling ratio analysis.* For evaluation of the water uptake by the materials, the thermo-responsive discs and three-dimensional scaffolds were analyzed for mass change after incubation at 25 °C and 37 °C (*n* = 4) in water. Each incubation interval was 24 h. At both times, the wet weights of the samples were recorded after excess water was absorbed by placing the samples on cellulose filter paper. Thereafter, the materials were freeze dried for 48 h before recording the dry weight. The swelling was calculated as follows:

$$\mathrm{Swelling}\;\mathrm{ratio}\;\left(g/g\right)=\frac{\mathrm{Weight}\;\mathrm{of}\;\mathrm{swollen}\;\mathrm{construct}\left(g\right)}{\mathrm{Dry}\;\mathrm{weight}\;\mathrm{of}\;\mathrm{construct}\;\left(g\right)}$$

*Delta swelling ratio:* In order to elucidate the changes in the swelling ratio at temperatures surrounding the temperature range of interest, the difference in the swelling ratios (g/g) determined at 37 °C and 25 °C was determined by subtracting the swelling ratio determined at 25 °C from the value determined after equilibration at 37 °C and reported as ΔSR (37/25).

ΔSR (37/25) = swelling ratio at 37 °C − swelling ratio at 25 °C

When thermo-gelation occurred in the covered temperature range, a negative value was expected for this parameter as water is expelled from the material when the temperature exceeds the phase transition temperature. A positive value indicated that swelling increased with incubation temperature and no phase transition affecting the swelling ration occurred between 25 °C and 37 °C.

*Rheological properties of printed constructs*. An oscillatory rheometer (MCR301, Anton Paar, Graz, Austria) was used to determine the responses of the selected three-dimensional scaffolds to changes in environmental temperature using the following analytical protocol: the equilibrium swollen samples were kept under constant normal force (0.2 N) with an 8 mm parallel plate geometry and analyzed in a temperature sweep experiment with an oscillation frequency of 1 Hz and an amplitude of 1% strain. After an equilibration time of 10 min, the temperature was ramped from 20 °C to 50 °C and vice versa with a heating/cooling rate of 3 K/min. Storage and loss moduli were recorded by the RHEOPLUS software (Anton Paar, Version 3.4).

*Differential scanning calorimetry (DSC).* The transition temperatures of the discs and scaffolds were determined by DSC (DSC1, Mettler Toledo, Greifensee, Switzerland). The samples were equilibrated in distilled water for 48 h and cut into small pieces, from which a sample was precisely weighted (10–15 mg) into a 40 µL aluminum crucible. A crucible filled with water (15 mg) was used as a reference. The heat flux DSC was performed between 10 and 60 °C with a heating rate of 3 °C/min. The first onset temperature of the recorded endothermic phase transition signal was designated as the transition temperature (T_trans_).

*Cell Culture Studies*. L929 fibroblasts and C2C12 myoblasts cells were cultured on printed scaffolds. Selected scaffolds were seeded with 2 × 10^5^ cells per construct in standard 12-well plates (Corning Inc., Corning, NY, USA). The plates were placed into an incubator at 37 °C for the cells to settle for two minutes before the wells were carefully filled with cell culture medium to completely cover the seeded constructs (2 mL). The cell culture medium (37 °C) was refreshed every 48 h after the temperature shift period was completed. A mechanical stimulation of the cells on the materials was induced through periodic temperature changes. In brief, after 24 h of cell seeding, the plates were taken out of the incubator (37 °C) and placed in the laminar air flow bench for 10 min to decrease the medium temperature. The plates were then placed in an incubator thermostat at 30 °C (5% CO_2_) for one hour before the plates were returned to the incubator at 37 °C. The periodic cycle was repeated every 24 h until the designated time point.

*Live/Dead Assay*. The viability of the cells on the material surfaces was qualitatively assessed by staining the cell-laden constructs with calcein-acetoxymethylester (calcein-AM) (λ_ex_ = 488 nm, λ_em_ = 520 nm) and ethidium homodimer-1 (λ_ex_ = 528 nm, λ_em_ = 617 nm) (both from Molecular Probes™, Invitrogen, Waltham, MA, USA) under light exclusion for 45 min. The live/dead staining solution was prepared by mixing 1 µL of calcein-AM and 2 µL of ethidium homodimer-1 with 2 mL of PBS. The samples were washed with PBS to remove excess dye and analyzed by fluorescence microscopy at λ_ex_ = 488 nm and λ_em_ = 520 nm for calcein in the live cells and at λ_ex_ = 528 nm and λ_em_ = 617 nm for ethidium homodimer-1 in the dead cells (Nikon DS-Ri2, Japan). Cell viability was documented at days 1, 3, and 7.

Live cell staining was quantified by analyzing the micrographs (TIFF format, recorded with an exposure time of 600 ms) (*n* = 3). The images underwent a registration process and three-dimensional reconstruction by Fiji software (ImageJ, NIH, Bethesda, MD, USA) [65]. The normalized fluorescence intensity (NFI) of each micrograph was calculated by averaging the fluorescence intensity (*I_a_*) and subtracting the average background intensity (*I_b_*) from a scaffold without a cell presence and dividing again by the average background intensity. The equation is shown below:

$$\mathrm{NFI}=\frac{I_{a}-I_{b}}{I_{b}\;}$$

## 4. Conclusions

This work presents thermo-sensitive scaffolds composed of TMPTA-cross-linked poly(NiPAAm-*co*-DMAEA-*co*-AMO) networks fabricated via photo-induced polymerization and three-dimensional cDLP printing methods from monomer resins. The resulting thermo-responsive disc/scaffold had a controllable swelling ratio and transition temperature. Simultaneously, as a printing solvent, we introduced glycofurol, which has good biocompatibility and improved the overall product printing quality. The formulation of 3% *w*/*w* TMPTA, N80-D15-A5, and 4% *w*/*w* PI, with an exposure time of 10 s per layer, was identified as the most promising for cell cultivation under periodic changes in the cultivation temperature at 36.3 °C ± 0.9 °C. This strategy provides an on-demand thermo-sensitive platform for cell mechano-stimulation and thus presents a promising alternative for the three-dimensional thermo-sensitive discs/scaffolds. These results suggest that the material platform should be further elucidated for controlled and cell type-adjusted mechanical stimulation so that it could finally be used for the engineering of a mechanical stimulation device for biomedical applications.

## Data Availability

Not applicable.

## Supplementary Materials

The following supporting information can be downloaded at: https://www.mdpi.com/article/10.3390/ijms24010572/s1.
