# Supplementary material for: Adjustable Thermo-Responsive, Cell-Adhesive Tissue Engineering Scaffolds for Cell Stimulation through Periodic Changes in Culture Temperature
Source: Int J Mol Sci. 2022 Dec 29;24(1):572. doi: 10.3390/ijms24010572 (PMC9820143; doi:10.3390/ijms24010572)
Supplement: Supplementary file 1 [file ijms-24-00572-s001.zip › ijms-1993661-supplementary.pdf]

Table S1. Formulations and abbreviated code as used for the fabrication of photo-polymerized discs.

Figure	Formulation Nx-Dy-Az (% w/w)	x	y	z	TMPTA addition (% w/w)
1A	N100-D0-A0	100	0	0	1, 5, 10, 20
	N95-D5-A0	95	5		
	N90-D10-A0	90	10		
	N85-D15-A0	85	15		
	N80-D20-A0	80	20		
1B	N80-D15-A5	80	15	5	5, 10
	N75-D15-A10	75	15	10	
1C	N70-D15-A15	70	15	15	1, 2.5, 5, 7.5, 10
	N60-D20-A20	60	20	20	
	N50-D25-A25	50	25	25	
2 (A, B, C)	N80-D15-A5	80	15	5	1, 2, 3
	N75-D15-A10	75	15	10	

Table S2. Formulations, processing conditions and abbreviated code as used for printed constructs by cDLP.

Figure	Formulation Nx-Dy-Az (3% w/w TMPTA)	%PI (w/w)	Exposure time/ layer (second)	Dilute solvent
3A	N80-D15-A5	2, 4	10, 20, 30	Ethanol
4A	N100-D0-A0	4	10	GF
	N85-D15-A0			
	N83-D15-A2			
	N80-D15-A5			
	N75-D15-A10			
	N70-D15-A15			
	N65-D15-A20			
4B	N100-D0-A0	4	10	GF
	N85-D0-A15			
	N83-D2-A15			
	N80-D5-A15			
	N75-D10-A15			
	N70-D15-A15			
	N65-D20-A15			

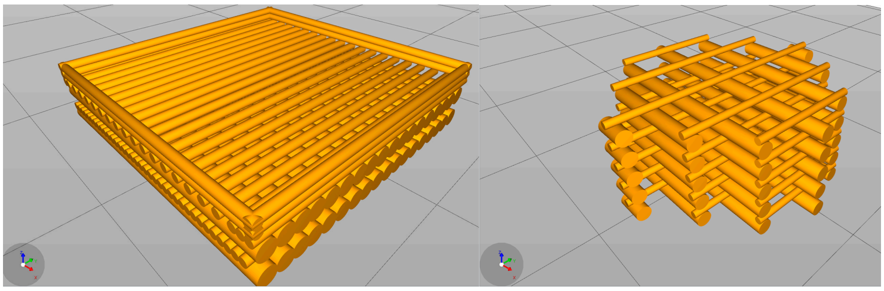

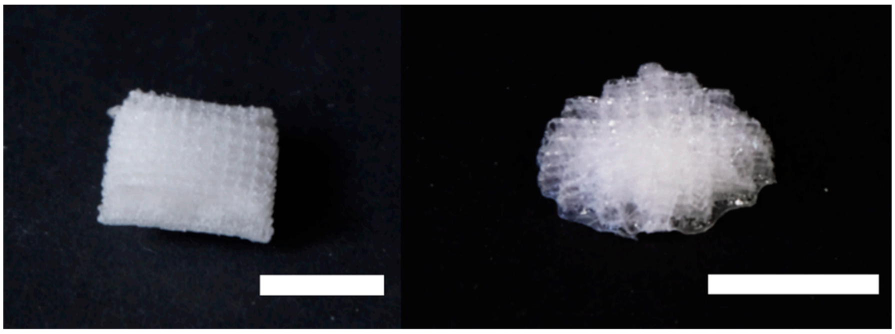

Figure S1. Visualization of the three-dimensional design and the printed and lyophilized structures (scale bar: 1 cm): raft (left) and three-dimensional lattice (right). The voxel sizes are following: 7.6 × 7.6 × 1.8 mm and 7.6 × 7.7 × 2 mm. The raft structure was designed to provide a well observable adhesion surface generated by side-by-side fibers, which do not allow the cells to penetrate into deeper layers. The three-dimensional lattice scaffold provides an open macroporous structure for three-dimensional cell adhesion and cultivation.

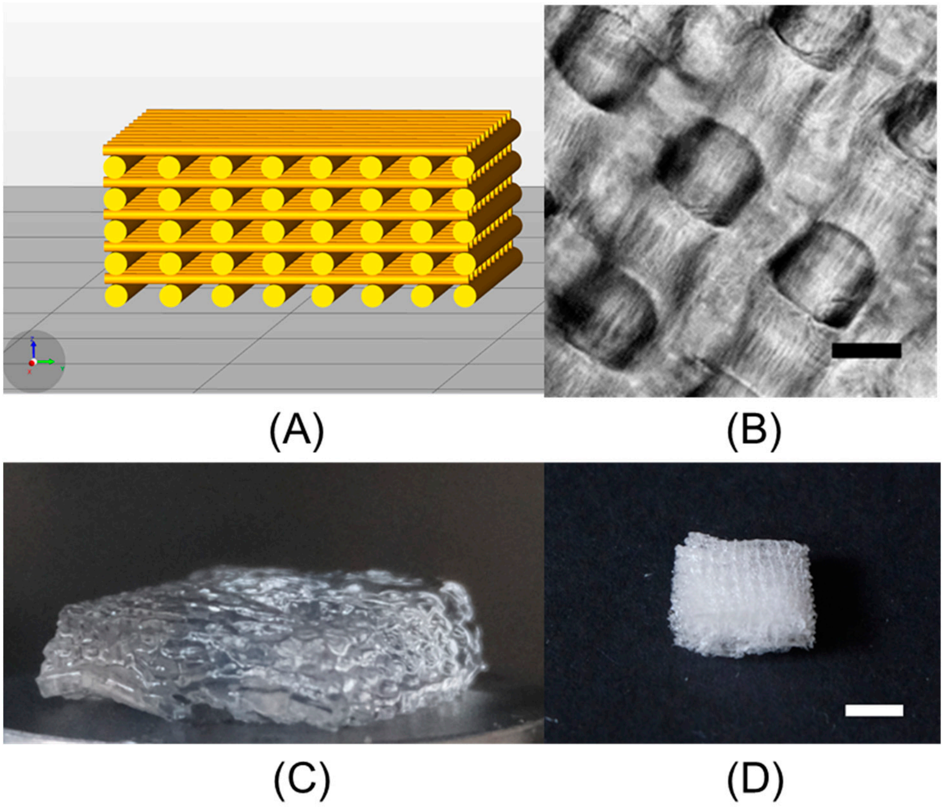

Figure S2. Images of three-dimensional lattice scaffolds (3% w/w TMPTA, N80-D15-A5). (A) Construction design (size: 7.6 x 7.8 x 3.4 mm), (B) top view of printed scaffold in equilibrium swollen state (scale bar: 500 µm), (C) hydrated structure and (D) lyophilized structure (scale bar: 5 mm).

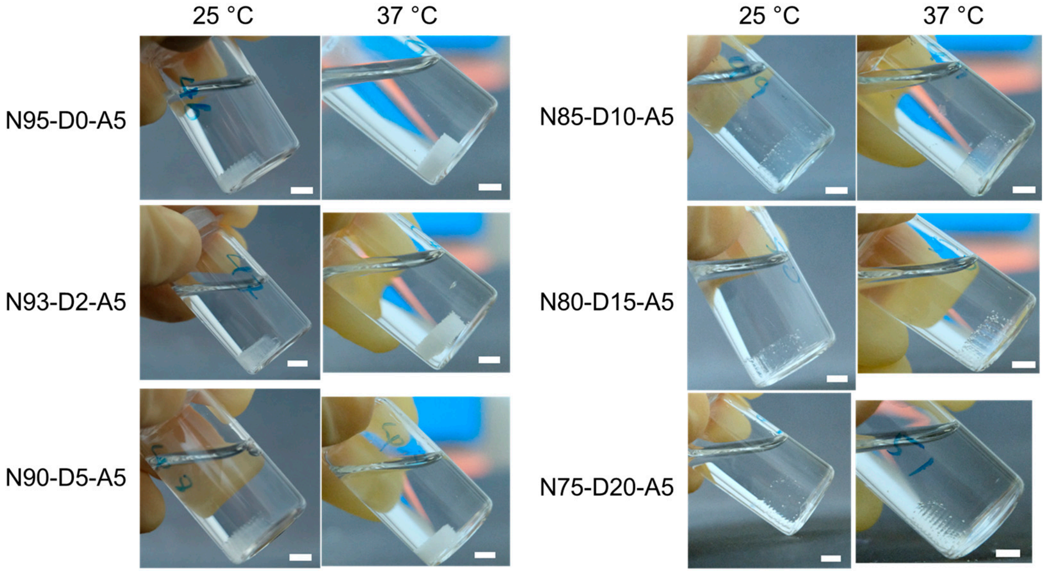

Figure S3. Thermo-responsive lattice type scaffolds fabricated from formulations with increasing DMAEA in PBS at 25°C (left) and 37°C (right). Resins were diluted with glycofurol and contained 3% w/w TMPTA, 4% w/w PI. The printing time was 10 sec/layer. Scale bar represents 5 mm. The result demonstrated the influence of the cationic molecules on phase transition of the scaffolds at different temperatures. Materials with lower DMAEA content and reduced transition temperature had an opaque appearance at 37°C which is indicative of scaffold dehydration. Scaffolds with higher DMAEA contents had a comparable appearance at 25°C and 37°C.

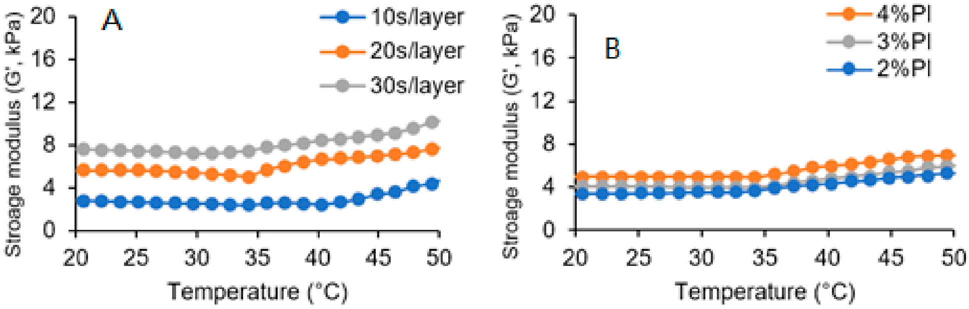

	Transition Temp. (°C)
**10 s/ layer**	33.7 ± 1.0
**20 s/ layer**	35.1 ± 0.8
**30 s/ layer**	35.5 ± 0.2
**4%PI**	35.3 ± 0.8
**3%PI**	35.4 ± 0.4
**2%PI**	35.8 ± 0.4

Figure S4. Rheological temperature sweep measurements from 20°C to 50°C (3 K min^{-1}) to elucidate the influence of (A) different printing times per layer (10, 20, and 30 sec/layer) and (B) photo-initiator ratio (2, 3, and 4% w/w) on storage modulus of printed lattice-type contracts (N80-D15-A5, 3% TMPTA, diluent: glycofurol) and. The bottom table summarized the transitional temperatures of the respective scaffolds as obtained from the DSC measurement. The determined moduli increased with exposure time per layer and photo-cross-linker contents. During the temperature sweeps the moduli increased when the transition temperature was passed due to increased polymer-polymer interactions and decreased hydration.

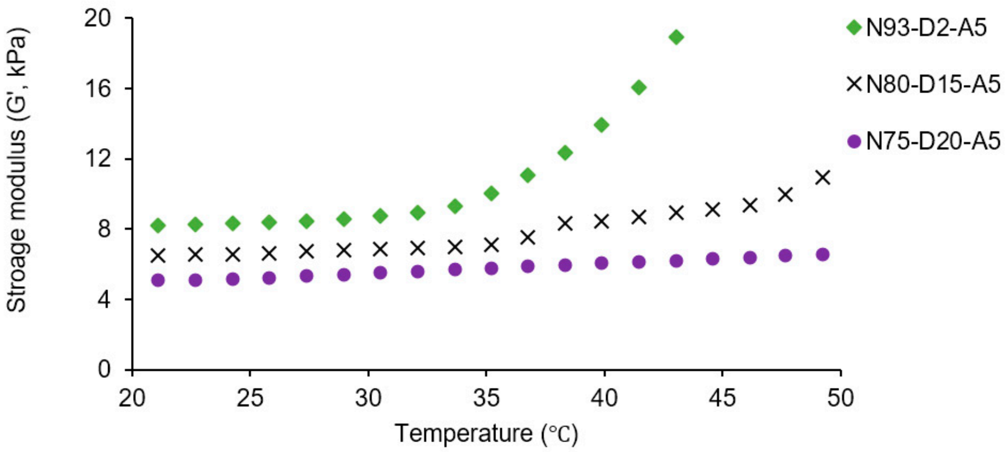

3%TMPTA	Transition Temp. (°C)
N93-D2-A5	33.0 ± 0.9
N80-D15-A5	35.3 ± 0.9
N75-D20-A5	37.6 ± 1.0

Figure S5. Oscillatory rheological storage modulus of printed 3% TMPTA scaffolds with different ratios of DMAEA (5% AMO, diluent: glycofurol, 4% Irg819, printing time 10 sec/layer). Temperature sweep at 1Hz and an amplitude gamma 1% performed from 20°C to 50°C and back down to 20°C with a ramp of 3 or -3 K min^{-1}. The transitional temperature of the scaffolds from the DSC measurement is shown in the table below. Increasing DMAEA content resulted in increased hydration, increased transition temperature and decreased moduli. In accordance, the phase transition-induced increase in storage modulus during the temperature sweeps was less pronounced in materials with higher DMAEA content.

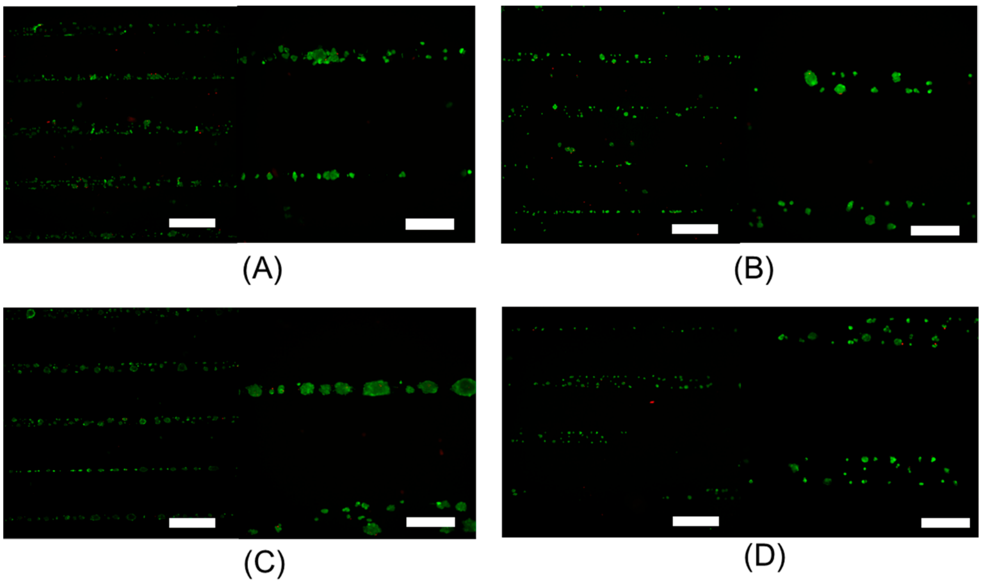

Figure S6. C2C12 of N80-D15-A5 scaffold at different time points ((A)1, (B)3, (C)5, and (D)7 days) cultivated at constant temperature (37°C). The cells showed no proliferation and low cell density. Scale bar represents 500 µm (left image of each pair) and 200 µm (right image of pair). Without cyclic temperature changes in cultivation temperature, no considerable cell proliferation was observed.
